# Is mass screening for coeliac disease a wise use of resources? A health economic evaluation

**DOI:** 10.1186/s12876-021-01737-1

**Published:** 2021-04-09

**Authors:** Fredrik Norström, Anna Myléus, Katrina Nordyke, Annelie Carlsson, Lotta Högberg, Olof Sandström, Lars Stenhammar, Anneli Ivarsson, Lars Lindholm

**Affiliations:** 1 Department of Epidemiology and Global Health, Umeå University, 901 87 Umeå, Sweden; 2 Department of Public Health and Clinical Medicine, Family Medicine, Umeå University, Umeå, Sweden; 3 Department of Pediatrics, Clinical Sciences, Skåne University Hospital, Lund University, Lund, Sweden; 4 Department of Paediatrics, Norrköping Hospital, Linköping University, Norrköping, Sweden; 5 Department of Clinical Sciences, Pediatrics, Umeå University, Umeå, Sweden

**Keywords:** Coeliac disease, Cost-effectiveness, Compliance, Long delay, Screening, Adolescent, QALY

## Abstract

**Background:**

Living with undiagnosed symptomatic coeliac disease is connected with deteriorated health, and persons with coeliac disease often wait a long time for their diagnosis. A mass screening would lower the delay, but its cost-effectiveness is still unclear. Our aim was to determine the cost-effectiveness of a coeliac disease mass screening at 12 years of age, taking a life course perspective on future benefits and drawbacks.

**Methods:**

The cost-effectiveness was derived as cost per quality-adjusted life-year (QALY) using a Markov model. As a basis for our assumptions, we mainly used information from the Exploring the Iceberg of Celiacs in Sweden (ETICS) study, a school-based screening conducted in 2005/2006 and 2009/2010, where 13,279 12-year-old children participated and 240 were diagnosed with coeliac disease, and a study involving members of the Swedish Coeliac Association with 1031 adult participants.

**Results:**

The cost for coeliac disease screening was 40,105 Euro per gained QALY. Sensitivity analyses support screening based on high compliance to a gluten-free diet, rapid progression from symptom-free coeliac disease to coeliac disease with symptoms, long delay from celiac disease with symptoms to diagnosis, and a low QALY score for undiagnosed coeliac disease cases.

**Conclusions:**

A coeliac disease mass screening is cost-effective based on the commonly used threshold of 50,000 Euro per gained QALY. However, this is based on many assumptions, especially regarding the natural history of coeliac disease and the effects on long-term health for individuals with coeliac disease still eating gluten.

**Supplementary Information:**

The online version contains supplementary material available at 10.1186/s12876-021-01737-1.

## Background

Coeliac disease affects approximately 1% of the population in most countries [1]. In Sweden the prevalence is higher, with reports of up to 3% [2, 3]. Coeliac disease is an autoimmune disease characterised by a permanent intolerance to gluten, causing small intestinal enteropathy in affected individuals. The only effective treatment is a strict gluten-free diet, which for most promotes recovery of the small intestinal mucosa [1]. Symptoms of coeliac disease include both gastrointestinal and extra-intestinal symptoms as well as diffuse symptoms, and even an experience of feeling symptom free for some individuals [4].

Many experience health-related problems before a coeliac disease diagnosis. A significant gain in health-related quality of life is associated with being diagnosed [5, 6]. The gain in mean value of the quality-adjusted life year score for coeliac disease patients has been shown in Sweden to improve from 0.66 before diagnosis to 0.86 after diagnosis [6], which is similar to the value for the general population in Sweden, and in the United Kingdom from 0.56, which the authors compared with the value for stroke patients, to 0.84 after diagnosis [5]. Furthermore, in both countries, most symptoms associated with coeliac disease improved for individuals after diagnosis [5, 7].

Coeliac disease is also associated with a higher prevalence of other autoimmune diseases such as diabetes type 1 [8] and thyroid diseases [9]. Diagnosis and the initiation of a gluten-free diet lower the risk of future complications, but the evidence for the effect varies [10, 11]. Increased mortality for those with coeliac disease has been reported irrespective of treatment or not, Tio et al. [12] reported in a meta-analysis a hazard ratio of 1.24, in which the Swedish study with the majority of the cases in their analysis had a hazard ratio of 1.39 [13]. The hazard ratio of the Swedish study have later been revised to 1.21 after extending the follow-up period from 2008 to 2017 [14]. Findings for coeliac disease occurrence and mortality are not conclusive and some studies show there is no evidence of an increased risk [15–17].

Because symptoms vary and because asymptomatic coeliac disease is common, the time to disease diagnosis can be long [4]. The median time from first symptoms to diagnosis varies between studies with recent reports ranging from 2 to 4 years [6, 18, 19]. Despite improvements in diagnostics and increased awareness, this delay has not shortened much over time [6]. Furthermore, screening studies have shown that most individuals with coeliac disease are undiagnosed at the time of screening, e.g. two of three cases being undiagnosed among Swedish 12-year-old children [3], four of five cases being undiagnosed among Swedish adults [20], and five–ten undiagnosed cases per diagnosed case in Western Europe [21].

As coeliac disease is a common disease with varying and diffuse symptoms there may be long delays to diagnosis and compromised health while waiting for the diagnosis. Mass screening could be an option to lower this burden, but due to insufficient evidence on the cost-effectiveness, amongst other things, there is no consensus that this is the best option [22, 23]. To determine the cost-effectiveness of a screening, improvements in health-related quality of life, as measured by quality-adjusted life years (QALYs) [24], must be compared to costs and savings. A few health economic evaluations of mass screening for coeliac disease have been conducted to determine the cost-effectiveness, and the most comprehensive are two done by Shamir et al. [25, 26], and one described by Park et al. [27]. The conclusion from these evaluations is that a mass screening for coeliac disease is likely to be cost-effective. However, there is a divergence between these evaluations, and the authors point out that within their analytical models some assumptions are based on weak evidence. Among other health economic evaluations of coeliac disease screening, the focus has usually been on at-risk groups [28–30]. Thus, a more comprehensive health economic evaluation, as conducted in this study, is imperative.

The aim of our study was to determine the cost-effectiveness of a coeliac disease mass screening at 12 years of age, taking a life course perspective on future benefits and drawbacks.

## Methods

### Study material

For most of the assumptions in our health economic evaluation, we relied on data from two study populations: (1) the Exploring the Iceberg of Celiacs in Sweden (ETICS) screening study [2] and (2) a survey among adult members of the Swedish Coeliac Association [6], referred to as “the adult coeliac disease survey”. The current study was planned in parallel with the ETICS screening study and the adult coeliac disease survey. Thus some of the data have been customised to also be used for this study, although the surveys mainly were created for previous publications [6, 7, 31–33]. The questionnaire for the adult coeliac disease survey is available online as additional file to a previous publication [6]. Despite relying on Swedish data, our model was not developed to be limited to the Swedish context.

ETICS was a school-based screening for coeliac disease among 12-year-olds in Sweden that was conducted during the school years 2005/2006 and 2009/2010 [2]. In the study, 13,279 children participated and 240 were diagnosed with coeliac disease. As part of the ETICS study, participating children and their parents were asked to fill in questionnaires in conjunction with the school-based screening prior to knowledge of the screening results. In the first field phase (2005/2006), we also sent a follow-up questionnaire to children with coeliac disease and controls among “healthy” children [32]. In the adult coeliac disease survey, a questionnaire was sent to 1560 randomly selected persons with coeliac disease who were members of the Swedish Coeliac Association in 2009, and 1031 (66%) participated [6].

In addition to these studies, we used data from the National Swedish Childhood coeliac disease register, referred to as the “coeliac disease register”, for one of our assumptions [34, 35]. This register captures all reported incident cases from paediatric clinics in Sweden. We also used official and publicly available statistics from Statistics Sweden and the Swedish Association of Local Authorities and Regions as well as results from scientific publications.

### The health economic evaluation

In our health economic evaluation, we used a Markov model with six health states, 1-year time cycles, and a lifetime horizon [36]. The model addressed the cost-effectiveness of coeliac disease screening of 12-year-olds in Sweden (screening alternative) in comparison with no screening for these children (no screening alternative). The Markov model was performed using a template in Microsoft Excel.

**Fig. 1:**
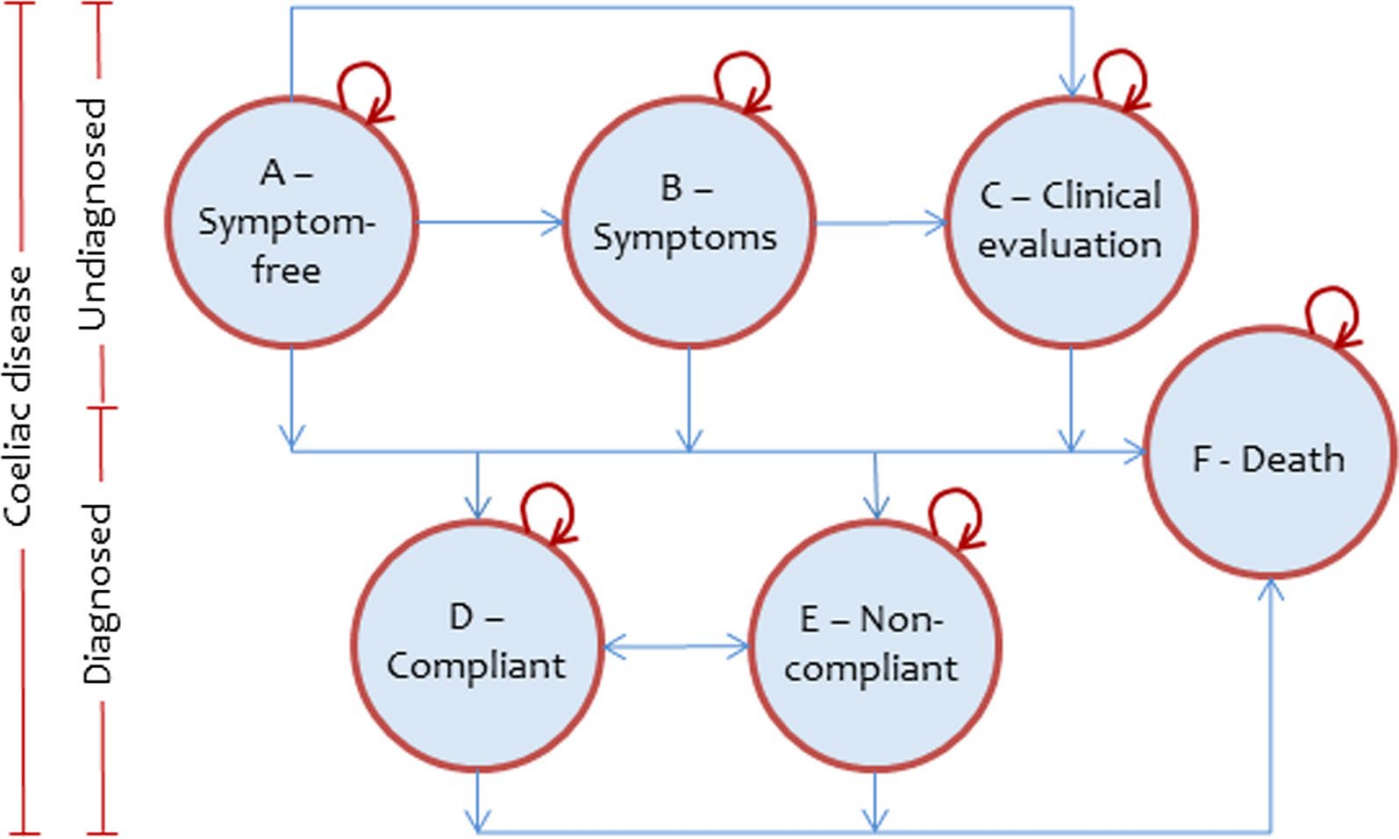
The health economic model

Figure 1 shows a flow chart for the health economic model with the five health states. The first three health states (A–C) refer to undiagnosed coeliac disease, the fourth and fifth (D and E) refer to having a coeliac disease diagnosis, and the last state (F) refers to death. The state “symptoms” (B) corresponds to the individual having persistent symptoms indicative of coeliac disease but without being assessed by a physician. The state”clinical evaluation” (C) corresponds to the individual having visited a physician to assess the symptoms without it yet resulting in a coeliac disease diagnosis. For state B and state C, we used different transition probabilities for the first year in the state and for later years by relaxing the Markov model [36]. The state “compliant” (D) refers to having a coeliac disease diagnosis and strict adherence to a gluten-free diet, while the state “non-compliant” (E) corresponds to having a coeliac disease diagnosis without strict adherence to a gluten-free diet.

Our proposed age for screening for coeliac disease is 12 years of age, which was the targeted age group in the ETICS screening study [2, 3]. In the ETICS study, children were referred to undergo a small intestinal biopsy if their serology demonstrated sufficient levels of antihuman tissue transglutaminase (atTG) and endomysial antibodies (EMA), both of type IgA and IgG (for IgA-deficient children). A coeliac disease diagnosis was given if the child either had a biopsy that revealed damage classified as Marsh 1 in combination with symptoms/signs compatible with coeliac disease or if they solely had a higher degree of small intestinal damage (Marsh 2–3c). For the screening strategy within the ETICS study, more details are available in section 1 of Additional file 1. For the screening alternative, we assumed that all children with coeliac disease were diagnosed during the screening. For the years following the screening and for all years in the no-screening alternative, individuals were diagnosed according to standard clinical practice.

We calculated the incremental cost-effectiveness ratio (ICER) and expressed it in terms of Euro per QALY gained. For both costs and QALYs, we used a 3% discount rate as recommended by the Dental and Pharmaceutical Benefits Agency in Sweden [37]. Our proposed screening was for the year 2017 due to data not being available for later years. We used the World Bank’s consumer price index for Sweden to recalculate costs to 2017 prices [38].

### Model assumptions

Our Markov model used assumptions in regard to (1) the initial distribution of individuals in the different states, (2) the transition probabilities between states, i.e. the conditional probability of being in a state the next year based on the current state (see arrows in Fig. 1), (3) the cost for a screening, (4) the cost for being in a state during a year, and (5) the QALY score for being in a state during a year. Time-dependent transition probabilities were only used for mortalities. We tested how these assumptions affected estimates of the ICER in sensitivity analyses. Complimentary descriptive analyses were conducted in Stata 13.1 (StataCorp LP, College Station, TX).

#### Initial distribution of individuals between the different states

The extent of symptoms during the past 6 months, as captured in the questionnaire by the children who were diagnosed with coeliac disease later, was used to decide on the distribution between states A–C at the start of our model. Based on this questionnaire, we assume that 3.8% of the children are in “symptoms” state, and 3.4% of the children are in “clinical evaluation” state, at the time of the screening. Consequently, most children in our model were assumed to have symptom-free coeliac disease (92.8% of them) at the time of our proposed coeliac disease screening. Additionally, we assume that 25% of those in state B had just developed their symptoms and 84% of those in state C were about to start an evaluation for coeliac disease. The reasoning behind these values are given in section 2 of Additional file 1. The assumptions are in line with information from the “adult coeliac disease survey” described in section 3 of Additional file 1.

#### Transition probabilities

Our transition probability matrix is shown in Table 1. We used different probabilities for the first year in states B and C compared to the following years.

**Table 1:** Transition probability matrix during a 1-year cycle

	A-Symptom-free	B-Symptoms	C-Clinical evaluation	D-Compliant	E-Non-compliant	F-Death
A	1–0.027–0.0090–0.0090–1.22*mr^a^	0.0027	0.0090	0.0090	0	1.22*mr
B	0	1–0.135^b^–0.450^c^–1.27*mr	0.135^b^	0.450^c^	0	1.27*mr
C	0	0	1–0.522^d^–1.27*mr	0.522^d^	0	1.27*mr
D	0	0	0	0.86 (1–1.22*mr)	0.14 (1–1.22*mr)	1.22*mr
E	0	0	0	0.86 (1–1.27*mr)	0.14 (1–1.27*mr)	1.27*mr
F	0	0	0	0	0	1

^a^mr = age-based mortality rate

^b^0.168 during the first year in the state

^c^0.734 during the first year in the state

^d^0.839 during the first year in the state

#### Transitions between states on a yearly basis

Data from the adult coeliac disease survey were used to derive estimates of the transitions from the non-diagnosed symptomatic states (B and C) on a yearly basis [6], which we explain in detail in section 3 of Additional file 1. For the transitions from the symptom-free state, we used the coeliac disease register and calculated the expected number of cases and fit this to our model [34, 35]. We assumed that ~ 50% of all cases would be diagnosed after 15 years in the no-screening alternative, which is consistent with the data in the coeliac disease register and previous publications [3, 20, 21]. For further details about this, see section 4 in Additional file 1.

#### Compliance to a gluten-free diet

In a recent publication using ETICS study data, the compliance rate was reported to be 86% based on a Swedish version of the Celiac Disease Adherence Test score developed by Leffler et al. [39, 40], and we used this value as the assumption in our model. We assumed that during each year this is the proportion of those with coeliac disease who are compliant to a gluten-free diet, while we assumed that all newly diagnosed coeliac disease cases are compliant during the first year. See section 5 in Additional file 1 for a more detailed explanation.

#### Mortality

We used age-based mortalities (for 12–14 years of age and thereafter in 5-year increments from 15 years of age, i.e. 15–19, 20–24, and so on) for the general population as presented by Statistics Sweden for the year 2017. Patients with coeliac disease, both undiagnosed and diagnosed, have commonly been assumed to have an increased risk of mortality [12, 13]. We assumed a hazard ratio of 1.27 for undiagnosed states (A–C) and the non-compliant state (E). For the compliant state (D) we assumed a hazard ratio of 1.22. See section 6 in Additional file 1 for a detailed explanation of the choice of hazard ratios.

#### QALY scores

In our model, we based our assumptions about age-related QALY scores on responses to the EQ-5D’s three-level descriptive system (EQ-5D-3L) [41], using the formula by Dolan et al. for adults in the United Kingdom [42]. For QALYs there are two anchor points, 0 (death) and 1 (full health), which enables comparisons of different health problems. The QALY scores were derived through complementary analyses using the ETICS-study (at the screening and follow-up) [31, 32], and the “adult coeliac disease survey” [6]. Our assumptions for the different states varied within age groups, from 0.81 to 0.94 for states A and D, from 0.69 to 0.87 for state B, from 0.56 to 0.78 for state C, and from 0.79 to 0.89 for state E. See section 7 in Additional file 1 for a detailed description of the derivation of utilities in our model and Table S1 in Additional file 1 for age-based QALY scores.

#### Costs

We used a societal perspective for estimating the costs, thus including costs for the health care sector in terms of health care visits and hospitalisation days (without subtracting patient fees), and costs related to productivity loss. In our cost perspective, we excluded costs for the individual (e.g. increased costs from buying gluten-free food) and for their family and friends, both in relation to the screening and to daily life with coeliac disease.

In a previous study by Norström et al. [33], the total cost for the ETICS screening in 2005/2006 was estimated to be 348,803 Euros. In a later phase of the ETICS-study, the number of confirmed coeliac disease cases increased [43]. Subsequently, we revised the cost to a total of 2589 Euros per diagnosed child, also recalculating costs to the 2017 prices.

The mean cost per health care visit was 312 Euros and the mean cost per hospitalisation day was 1137 Euros based on information from the Swedish Association of Local Authorities and Regions for the year 2017. The cost for productivity loss (working ages 19–65 years) was assumed to be 230 Euros per day, which corresponds to the average daily income (average income of 3370 Euros per month divided by 22 working days per month) as reported by Statistics Sweden for 2017 [44], multiplied by a payroll tax of 50% (a representative rate in the Swedish labour market). During school years (≤ 18 years of age) and after retirement (> 65 years of age), no productivity loss was assumed. The use of health care, i.e. health care visits and hospitalisation days, and days of illness for each state were mainly based on the adult coeliac disease survey [7]. See section 8 in Additional file 1 for a detailed description of our assumptions presented in Table S2 in Additional file 1. For each state in the Markov model, the cost items were multiplied by the frequency per year in which they were utilised (Table 2).

**Table 2:** Costs, frequency of health care use, and days of illness before (states A–C) and after (states D and E) having coeliac disease diagnosis

	Health care visits		Hospitalisation days		Days of illness	
Cost per visit (euro)	311.9		1136.9		153^a^	
Age group^b^	≤ 65	> 65	≤ 65	> 65	≤ 65	> 65
Frequency for state						
A—Symptom-free	3.7	5.1	0.7	1.0	2.5	0
B—Symptoms	3.7	5.1	0.7	1.0	3.7	0
C—Clinical evaluation	5.4	5.4	2.3	2.9	7.2	0
D—Compliant	3.7	5.1	0.7	1.0	2.5	0
E—Non-compliant	4.5	5.3	1.5	2.0	4.9	0

^a^A cost of 0 euro is assumed for ages 12–18 years

^b^Presented for up to 65 years of age and above 65 years of age

### Sensitivity analysis

We performed sensitivity analyses by alternating one key parameter at a time (case 1 through 7) in our model:

Doubling the cost of the screening.
A sensitivity for atTG IgA of 95%, and a sensitivity for atTG IgG of 98.7% [25, 45]. Additionally, 1.3% of the children with coeliac disease were assumed to have IgA deficiency [3, 43].
(a) Compliance to a gluten-free diet of 96%, which is in accordance with surveys in patient organisations [6, 46], and (b) compliance to a gluten-free diet of 78%, which is the median adherence rate in a recent systematic review [47].
(a) No increase in mortality risk for those with coeliac disease undiagnosed or diagnosed, i.e. a hazard ratio of 1, (b) a hazard ratio of 1.60 for both diagnosed and undiagnosed, which was assumed by Hershcovici et al. [25], and (c) a hazard ratio of 1.60 for states A–C and E and 1.10 for state D.
(a) Doubling the transition probabilities from symptom-free (state A) to states B–D, and (b) halving the transition probabilities from symptom-free (state A) to states B–D.
Halving the transition probabilities to diagnosis (state D) for symptomatic undiagnosed states (states B and C).
Reducing the utilities for states A and B by 0.05.

## Results

The ICER for a coeliac disease screening was 40,105 Euro per gained QALY with a discount rate of 3% and 31,948 Euro per gained QALY with no discount (Table 3). Thus the ICER was below the commonly recommended cost-effectiveness threshold of 50,000 Euro per gained QALY.

**Table 3:** Cost-effectiveness of a coeliac disease screening – base case and sensitivity analyses

	Screening		No screening		Incremental costIncremental cost		ICER
	Cost	QALY	Cost	QALY	Cost	QALY	
Base case							
Discount	76,620	24.70	72,264	24.60	4356	0.11	40,105
No discount	179,858	57.45	174,236	57.27	5622	0.18	31,948
Sensitivity analyses^a^							
(1) Double cost of screening	79,209	24.70	72,265	24.60	6944	0.11	63,490
(2) Not all cases diagnosed in screening	76,532	24.69	72,265	24.60	4267	0.10	42,874
(3a) Compliance of 96% to gluten-free diet	72,375	24.86	69,831	24.68	2544	0.18	14,068
(3b) Compliance of 78% to gluten-free diet	80,015	24.58	74,211	24.53	5805	0.05	114,212
(4a) No increase in mortality due to CD	77,347	24.94	73,011	24.84	4336	0.10	42,764
(4b) Hazard ratio of 1.60 for all states	75,594	24.38	71,263	24.37	4331	0.10	42,440
(4c) Hazard ratio of 1.60 for states A, B, C, and E and 1.10 for state D	76,794	24.76	72,248	24.59	4546	0.17	26,692
(5a) Doubling transition probabilities from state A to states B, C, and D	76,620	24.70	73,414	24.52	3206	0.19	17,278
(5b) Halving transition probabilities from state A to states B, C, and D	76,620	24.70	70,992	24.68	5629	0.03	195,366
(6) Halving the transition probabilities to diagnosis for symptomatic undiagnosed states	76,620	24.70	74,172	23.93	2448	0.78	3145
(7) 0.05 lower QALY scores for states A and B	76,620	24.70	72,265	24.00	4356	0.71	6172

*ICER* incremental cost-effectiveness ratio, *QALY* quality-adjusted life year. Costs are measured in euros

^a^A discount of 3% for costs and QALYs are assumed for all sensitivity analyses

In our sensitivity analysis, we found a higher compliance rate to a gluten-free diet (case 3a), a faster progression from the symptom-free coeliac disease state (case 5a), a slower progression from symptoms to diagnosis (case 6), and poorer health for undiagnosed coeliac disease cases (case 7) heavily favoured the screening alternative (Table 3). Conversely, a poor compliance rate (case 3b) and a slower progression to symptomatic states (case 5b) supported the no-screening alternative. Doubling the cost for the screening (case 1) affected whether the cost per QALY for a screening was above or below the cost-effectiveness threshold, but the effect on the cost per QALY was not extensive. A large gap in standardized mortality risk between the compliant state, assuming hazard ratio of 1.10, and other states, assuming a hazard ratio of 1.60 (case 4c), affected the cost per QALY to some extent in favor of a screening. Other assumptions of the standardized mortality risk (case 4a and 4b) had a negligible effect on the ICER.

## Discussion

We have performed the most comprehensive cost-effectiveness analysis of a coeliac disease mass screening to date. Our study suggests that coeliac disease screening at the age of 12 years, at least in the Swedish context, can be considered cost effective based on the usually agreed threshold of 50,000 Euros per QALY.

Considering the difficulties in obtaining crucial information for our health economic evaluation, there are still questions to be answered. Some assumptions in our model are more sensitive than others to deviations. For example, a screening is highly likely to be cost-effective if patients comply with a gluten-free diet, and in our study we assumed a relatively high compliance (86%), which is the case in many countries [47]. In studies performed with Swedish and Finnish patient organisations, the compliance rate was even higher than we assumed in our study [6, 46]. Obviously, it is important to plan for adequate support for coeliac disease patients to be able to maintain a strict gluten-free diet before screening is considered [5, 6, 18, 19].

Several aspects of the disease development and diagnostic process are of importance if a screening is to be recommended. A screening is more likely to be cost-effective if the progression from symptomatic disease to diagnosis is characterised by a long patient and/or physician delay, if those with coeliac disease do not remain symptom-free for a long period of time, and if the QALY scores in the undiagnosed states before clinical evaluation are low. While screening has not yet been put in practice, decreasing the delay to diagnosis would be beneficial. Our findings corroborate that understanding the natural history of coeliac disease is important for determining the cost effectiveness of a coeliac disease mass screening. Previous studies have shown that those with a screening detected coeliac disease diagnosis have similar health as those in the general population [31, 48, 49], and thus our assumptions of similar QALY scores for symptom free and compliant states are reasonable. If these health consequences are underestimated, a screening would be favored.

Our cost-effectiveness analysis presents a similar conclusion as the studies by Shamir et al. and Park et al. [25–27]. Still, there are fundamental differences in our modelling, which have also led to differences in interpretations of key components in the models. Shamir et al. highlighted the increased mortality due to coeliac disease and the prevalence of coeliac disease as two key factors for their model [26], while in our analysis different characteristics were more important. In the study by Hershcovici et al. [25], the delay to diagnosis was a key factor for their conclusions, which was also relevant for our model. In comparison with the cost-effectiveness analysis by Herchcovici et al. [25], as well as that by Park et al. [27], we did not use different states based on extraintestinal symptoms or complications. This would have been valuable, but due to a lack of data we did not consider it beneficial to add such states to our model.

The major strength with this study is that we have been able to build on previous health economic evaluations of coeliac disease mass screening and have provided new information based on the ETICS study—which is the most comprehensive coeliac disease mass screening performed among the general population of children with over 13,000 participants. In addition, we have used results from a survey of adults in the Swedish Coeliac Association. However, making long-term assumptions in our evaluation, e.g. the consequences of late diagnosis or long-term health effects, has been challenging due to a lack of previous studies. Longitudinal prospective studies that follow individuals with coeliac disease, with and without a gluten-free diet, would increase knowledge on the natural history of both treated and untreated coeliac disease. For the best evidence, a randomised controlled study would be needed, but in most cases such a study would not be ethically acceptable. In observation studies, it is difficult to determine the potential protective effects of a gluten-free diet, and thus many of the assumptions related to the compliance and treatment effect are bound to be built on weaker evidence.

A strength in our study is that we were able to assume QALY scores from responses to the EQ-5D-3L instrument for individuals at all ages. We used Dolan’s formula to derive QALY scores, which was constructed for adults in the United Kingdom. This formula has been frequently used for the Swedish adult population and is likely to work well for adults in our study. However, for children the formula might bias our estimates, and this is a weakness that is unavoidable until a formula specifically for children is developed. Contrary to Shamir et al. [26], but in line with Hershcovici et al. [25], we limited ourselves to evaluating one screening strategy, namely the one that was used for the first field phase of the ETICS study [3]. This screening strategy included screening for IgA deficiency. Despite the increased risk for coeliac disease among those with IgA deficiency [1], only a few cases were referred to a biopsy in this group. Thus, the cost-effectiveness is likely only slightly different for other strategies.

One limitation is that we do not present gender-based analyses. This is due to the difficulty in achieving specific estimates for both men and women, with the exception of gender-based mortalities. In our study we propose a screening of 12-year-olds, who were mostly 13 years at the time of diagnosis. This is a sensitive age, and screening at a different age might be valuable to investigate in a future study. Another limitation may be that we have excluded costs for the individual (e.g. increased costs from buying gluten-free food) and their family and friends, both in relation to the screening and to daily life with coeliac disease. It has been shown that the gluten-free diet is increasing the cost [50], but at least from the Swedish perspective the actual cost have not been investigated further. Due to lack of data for the Swedish context and thereby uncertainties about the factual increased cost, as well as uncertainties about future differences in costs between gluten-free diet and other alternatives, we decided to not include this cost in our evaluation. It is also difficult to find accurate estimates for other assumptions about costs for family and friends, which probably explains why the consequences for people close to patients are rarely included in health economic evaluations. It cannot be ruled out that these costs and burdens could have affected our results, but we expect such effects to be small.

Some of our assumptions can be considered context specific. This will be important to take into account when evaluating the cost-effectiveness of a screening in other countries. The compliance rate varies geographically and is an important component for the cost-effectiveness as seen in our sensitivity analyses [47]. The prevalence of coeliac disease also varies [1], but even if our assumed prevalence is halved the screening cost will only increase by 52%, which does not have a substantial effect on the cost-effectiveness in our analyses. For some assumptions, previous studies have shown similar results in Sweden and other countries, e.g. the delay from symptoms to diagnosis [6, 18, 19], QALYs and appearance of symptoms before and after a coeliac disease [5, 6], and the increase in mortality related to coeliac disease [12]. Our health economic evaluation can therefore offer guidance for the implementation of screening of children for coeliac disease in several other countries.

Our model mainly used information from clinically detected cases, while we were evaluating the future for screening detected cases. In a study by Kivelä et al. [51], screened and clinically diagnosed cases showed similar enteropathies, and similarities between these groups have also been shown for different health indicators by the same research group in a long-term follow-up [52]. We therefore trust that our analyses are valid despite the lack of follow-up data for screened individuals.

One key aspect of a screening is how life is affected by living with a diagnosis, both in terms of protecting against future health deterioration and in coping with life-long treatment. For coeliac disease, there is varying evidence for a substantial negative impact on future health in addition to symptoms and nutritional deficiencies by eating a gluten-containing diet both in terms of other autoimmune disease and a shorter lifespan [4, 10, 11]. Regarding mortality, some studies have indicated no increased risk [15–17], while others generally have presented a rather modest effect [12, 13]. The benefits of a gluten-free diet can therefore be discussed in relation to the value of an earlier diagnosis for symptom-free coeliac disease patients. The challenges of living with coeliac disease are well documented, so whether the disease-burden is outweighed by the benefits for all individuals with coeliac disease is questionable [1]. Consequently, the gain of a coeliac disease mass screening may be lower than our study shows.

## Conclusion

In conclusion, according to the thresholds for cost-effectiveness, a coeliac disease screening can be recommended. For populations who are likely to have a high compliance to a gluten-free diet, the value of a coeliac disease screening is high. With proper early detection of coeliac disease, the value of a coeliac disease mass screening is lower. However, it should be noted that this is based on many assumptions, especially regarding the natural history of coeliac disease and the effects on long-term health for individuals with coeliac disease (diagnosed or not) eating gluten.

## Supplementary Information

**Additional file 1.** Appendix.

## Data Availability

The datasets generated and/or analyzed during the current study are not publicly available because the Swedish Data Protection Act (1998:204) does not permit sensitive data on humans (like in our interviews) to be freely shared. The datasets are available based on ethical permission from the Regional Ethical board in Umeå, Sweden, from corresponding author (Fredrik Norström). The Excel sheet for the health economic evaluation is available upon request.

## Abbreviations

EQ-5D: EuroQol 5 dimensions
EQ-5D-3L: EuroQol 5 dimensions three-level descriptive system
ETICS: Exploring the Iceberg of Celiac Disease
ICER: Incremental cost-effectiveness ratio
QALY: Quality-Adjusted Life-Year
