# Supplementary material for: Is mass screening for coeliac disease a wise use of resources? A health economic evaluation
Source: BMC Gastroenterol. 2021 Apr 9;21:159. doi: 10.1186/s12876-021-01737-1 (PMC8034082; doi:10.1186/s12876-021-01737-1)
Supplement: Supplementary file 1 — Additional file 1. Appendix. [file 12876_2021_1737_MOESM1_ESM.docx]

# Additional file 1

## The diagnostic procedure within the ETICS study

The criteria for children within the ETICS study to be referred for a small intestinal biopsy were related to values of antihuman tissue transglutaminase (atTG) of immunoglobulin types A (IgA) and G (IgG) in accordance with the manufacturers’ instructions (Celikey, Phadia GmbH, Freiburg, Germany), total serum-IgA (s-IgA) in accordance with the manufacturers’ instructions (BN Pro Sce System, Dade Behring, Marburg GmbH, Germany), and endomysial antibodies (EMA) of isotype IgA and IgG in accordance with the manufacturers’ instructions (The Binding Site, Birmingham, United Kingdom).

Those referred to biopsy fulfilled any of the criteria below:
i) atTG IgA above 4 U/mL.
ii) intermediate values for tTG IgA (2-4 U/mL) in combination with a positivity for endomysial antibodies (EMA) of isotype IgA.
iii) s-IgA below 0.06 g/L and atTG IgG above 6 U/mL.
iv) s-IgA below 0.06 g/L, intermediate values of atTG IgG (3-6 U/mL), and EM-IgG positivity.

The criteria for a coeliac disease diagnosis in the ETICS study were either Marsh 1 in combination with symptoms or signs compatible with coeliac disease or a higher degree of small intestinal damage. A more thorough description of the screening strategy has been published elsewhere [1] .

## Assumptions for initial distribution between the different states

The extent of symptoms during the past six months for children who were diagnosed with coeliac disease later, as captured by the questionnaire, was used to decide on the distribution between states A–C at the start of our model. The symptoms relevant for the model were those that the children would later understand as indicative of coeliac disease. For this purpose, we used reports of persistent abdominal or gastrointestinal symptoms, namely poor appetite, nausea, stomach-ache, upset stomach, abdominal gas, bloating, loose stool, and hard stool. Response alternatives “often” and “always” were considered as having persistent symptoms, while the other responses (“never”, “seldom”, “sometimes”) were considered as non-persistent symptoms. The symptoms upset stomach, abdominal gas, and distended stomach were merged as “bloated”, and the symptoms hard and soft stool were merged as “altered stool”, leading to a total of five symptoms. Disclosing two symptoms was defined as “symptoms”, which 3.8% (9/236 respondents) of the children reported. Claiming at least three symptoms was defined as “clinical evaluation”, which 3.4% (8/236 respondents) reported. We assumed that 25% of those in state B had just developed their symptoms and 84% of those in state C would have just started their evaluation for coeliac disease. These assumptions are in line with information from the “adult coeliac disease survey” described in the next section in this Appendix. Consequently, most children in our model (92.8%) were assumed to have symptom-free coeliac disease at the time of our proposed coeliac disease screening.

## Assumptions for transitions from non-diagnosed symptomatic states

We used the adult coeliac disease survey to derive transition probabilities for the non-diagnosed states. For the year after first symptoms of coeliac disease, 73% (602 of 820) of the participants were diagnosed within a year and 63% (125 of 199 who responded to this question) of those not diagnosed visited the doctor during this year. Thus, 17% were visiting a doctor without getting a diagnosis during the first year after the onset of symptoms. For the second year after the initial symptoms, these numbers were 45% (98 of 218) and 12.5% (10 of 74), and we used this for year two and beyond in our model for the transitions from state B. We used the same reasoning for transitions from state C to D, where we considered that 84% (717 of 855 participants) were diagnosed within a year from first meeting the doctor and that 52% (72 of 138) of those not diagnosed within the first year had their diagnosis during the second year after meeting with the doctor for the first time. These transitions were used for all years in the no-screening alternative, while they were used from the end of year one in the screening alternative, i.e. the year of the suggested screening.

For the year of the screening, we assumed that all were diagnosed in our base case, while we assumed a lower rate in our sensitivity analyses. In the sensitivity analysis, we assumed the percentages of sensitivities for non-diagnosed states for ttG IgA to be 95% and for tTG IgG to be 98.7%. For tTG IgG this sensitivity was assumed among the 1.3% of the children with assumed IgA deficiency. This resulted in approximately 95% of the children being diagnosed at the end of year one if they are screened.

The sensitivities was used in the previous health economic evaluation by Hershcovici et al. [2], and are motivated by a review conducted by the American Gastroenterological Association [3]. In the ETICS study, the threshold for positive serology was lower than the recommendation, meaning that the sensitivity of the test procedure is likely even higher for our proposed screening [1]. We choose this lower level to increase confidence in our assumption of misdiagnoses based on serology in the screening. The proportion of children with IgA deficiency in our sensitivity analysis was based on the first field phase of the ETICS study, were two of 153 children had IgA deficiency [1, 4].

## 4. Assumptions for transitions from a symptom-free state

To make an assumption about the probability of being clinically detected, we used the National Swedish Childhood coeliac disease register [5, 6]. In the register, there were 149 new coeliac disease cases among children born in 1993 during their 12^th^ and 13^th^ year compared to the 31.2 cases expected when compared with the number of cases among those born in 1987–1992 at these ages. In ETICS, ~16.1% (7,500 of 46,299) of all children born in 1993 without a coeliac disease diagnosis participated, and therefore only 4.1% (0.161 × 31.2 / (149 − 31.2) + 0.161 × 31.2) – i.e. the expected cases in the ETICS study area divided by the “added” extra cases attributed to the screening and the expected cases among these children if no screening – of those diagnosed within the ETICS study would be expected to be diagnosed during the year of the screening if the screening had not taken place. We used this information for our assumptions of the transitions from symptom free to other states. We assumed that 50% would be diagnosed after 15 years, which gave us a yearly transition of 4.5% of the asymptomatic coeliac disease cases to either diagnosis or a symptom state. We assumed that 60% would move to a symptom state, 20% to an evaluated state, and 20% to a diagnosed state on a yearly basis. Based on these assumptions and the assumptions about the initial distribution of children at age 12, i.e. the time of screening, we had 5.5% of the children diagnosed if no screening during the first year and thereafter around 4.8% of the undiagnosed cases on a yearly basis, which is higher than the 4.1% that we calculated as expected cases during the year of the screening. We consider 4.1% to be somewhat low because not all cases are reported to the register, and we find the higher incidence of new coeliac disease diagnoses to be more realistic.

## 5. Assumptions for compliance to a gluten-free diet

In a systematic review of adherence to a gluten-free diet by Hall et al. from 2009, the compliance rate was reported to range from 42% to 91% [7]. In the Nordic countries, the compliance rate is generally reported to be higher than in other countries [8]. In Viljamaa et al., the gluten-free diet compliance rate among Finnish adults was 93% for symptomatic cases and 96% for screening-based cases [9]. In the adult coeliac disease survey, the compliance rate was 96% among both symptomatic and screened patients [10]. Other studies also indicate that compliance is similar among symptomatic and screening-based cases. In the 5-year follow-up during the second field phase of the ETICS study, it was shown that 94% of the children were complying with a gluten-free diet at least “often”, but when using the Celiac Dietary Adherence Test – developed to measure gluten-free diet compliance by Leffler et al. [11] – the adherence decreased to 86% [12]. We therefore assumed that 86% of the children diagnosed would adhere to the diet. We used the same assumption for the other ages because the compliance rate from the survey data was from Swedish adults who were members of the Swedish Coeliac Society and therefore more likely have higher compliance than those with coeliac disease in the population who are not active in this group. We also assume that all newly diagnosed cases are compliant during the first year of diagnosis.

## 6. Assumptions for mortality

Tio et al. derived a pooled odds ratio of 1.24 for coeliac disease patients compared to the general population in a meta-analysis [13]. The largest study within their analysis was the study by Ludvigsson et al. [14], which was based on Swedish biopsy data between 1969 and 2008. That study presents an overall hazard ratio of 1.39 (comparing those diagnosed with coeliac disease with healthy controls), but due to the mortality risk being considerably larger in the first year after diagnosis, the hazard ratio was 1.22 for one–five years and 1.27 for more than five years after the coeliac disease diagnosis. The Swedish study follow-up in 2017 found a hazard ratio that was 1.21 [15]. Among studies in Tio et al.’s meta-analysis, some showed no signs of increased mortality for those with coeliac disease compared to the general population [16, 17], which was also shown in a recent study by Abdul Sultan et al. in the United Kingdom [18]. Our assumed increased mortality due to coeliac disease was also lower than the more recent studies by Holmes and Muirhead, which presents an SMR of 1.57 [19], and Quarpong et al., which presents an SMR of 1.43 [20]. However these studies, as well as others, relied on fewer cases than the study by Ludvigsson et al. [14].

In our model we used the hazard ratio 1.27 based on the study by Ludvigsson et al. for both the undiagnosed symptomatic states and the non-compliant state. For the compliant state (state D), we assumed a lower relative risk of 1.22 because previous publications indicate that treated coeliac disease lowers the mortality risk, which is evidenced by a decreased risk more than one year after diagnosis. Universal screenings for coeliac disease were introduced only three decades ago [21-24], and there is a lack of evidence based on comparisons between screened and unscreened coeliac disease in regards to mortality. Our estimates for undiagnosed coeliac disease are lower than in the health economic evaluation by Herchcovici et al. [2], which showed a relative risk of 1.60, but they might have revised their assumptions if they could have considered our more recently published studies. In a sensitivity analysis, we used the relative risk of 1.60 for this reason.

## 7. Derivations of health utilities

For the age group 12–14 years, the screening-detected children in the ETICS study were used. For assumptions of utilities in states A–C, we used the mean QALYs for the same groups as previously explained in the section “Initial distribution of individuals in the different states”. The mean QALYs were 0.94 (n = 190, standard deviation (SD) 0.12) for state A, 0.87 (n = 33, SD 0.15) for state B, and 0.74 (n = 10, SD 0.30) for state C. For state D we used the mean QALY of 0.94 (n = 111, SD 0.12) at the one-year follow up (Table 2).

For the age groups 20–24 years and older, utilities were based on the adult coeliac disease survey. We used the mean QALYs for the year before diagnosis – using the participant’s age at diagnosis – for state C. For state D, the age-based mean QALYs on the day of filling in the questionnaire, when most were compliant with a gluten-free diet, were used for the age groups 20–24 and older. In the study by Norström et al. [10], the mean QALY was similar for the general population and adults with coeliac disease, so we therefore used the same utility values for states A and D. We lacked information about current health for those aged 15–19 and for the states in our model, so we therefore used the average of the utility value for 12–14 years and 20–24 years. Due to the lack of a good source, we decided to add 0.13 to the QALYs for state C and state B for all ages, with the exception of states where the utility value would have been too close to the utility for state A, in which case the mid-point between state A and C was used. Only a few of the participants in the adult coeliac disease survey were non-compliant, and because we lacked other reliable sources for state E we used the utility value for state C and added 3/4 of the difference between this state and state D.

**Table S1.** Age-based QALY scores

|  | **State** | | | | |
| --- | --- | --- | --- | --- | --- |
| Age | Symptom-free | Symptoms | Evaluation | Compliant | Non-compliant |
| 12-14 | 0.94 | 0.87 | 0.74 | 0.94 | 0.89 |
| 15-19 | 0.92 | 0.82 | 0.69 | 0.92 | 0.86 |
| 20-24 | 0.90 | 0.76 | 0.63 | 0.90 | 0.83 |
| 25-29 | 0.87 | 0.76 | 0.63 | 0.87 | 0.81 |
| 30-34 | 0.85 | 0.76 | 0.58 | 0.85 | 0.78 |
| 35-39 | 0.89 | 0.69 | 0.56 | 0.89 | 0.81 |
| 40-44 | 0.85 | 0.74 | 0.61 | 0.85 | 0.79 |
| 45-49 | 0.86 | 0.80 | 0.67 | 0.86 | 0.81 |
| 50-54 | 0.87 | 0.87 | 0.74 | 0.87 | 0.84 |
| 55-59 | 0.87 | 0.84 | 0.71 | 0.87 | 0.83 |
| 60-64 | 0.83 | 0.81 | 0.78 | 0.83 | 0.82 |
| 65-69 | 0.86 | 0.81 | 0.76 | 0.86 | 0.83 |
| 70-74 | 0.85 | 0.80 | 0.74 | 0.85 | 0.82 |
| ≥75 | 0.81 | 0.81 | 0.81 | 0.81 | 0.81 |

## 8. Assumptions for utilisation of health care visits

The utilisation of health care visits, hospitalisation days, and sick days for each state were mainly based on the adult coeliac disease survey [25]. In the adult coeliac disease survey, it has previously been reported that respondents had an average of 5.4 health care visits, 2.3 hospitalisation days, and 7.2 sick days the year before diagnosis (used for state C) and 3.7 health care visits, 0.7 hospitalisation days, and 2.5 sick days during the year preceding the questionnaire (used for states A and D). In the adult coeliac disease survey, the health care utilisation was higher for participants 65 years and older, and we adjusted the utilisation for these years for these states in line with these survey data. For state B, individuals were assumed to not have met the physician due to coeliac disease related symptoms, and therefore had the same health care utilisation as in states A and D. However, we assumed a higher number of sick days, namely 3.7 days per year. For the non-compliant state, we assumed an utilisation in between the values for state C and state D as a consequence of not strictly following the diet. The total costs for each state and age are shown in Table 2 below.

**Table S2.** Annual costs per state and age group in Euros.

| State | 0–18 years | 19–65 years | 66 years and older |
| --- | --- | --- | --- |
| A – Symptom-free | 1,950 | 2,332 | 2,387 |
| B – Symptoms | 1,950 | 2,516 | 2,387 |
| C – Clinical evaluation | 4,299 | 5,401 | 4,299 |
| D – Compliant | 1,950 | 2,332 | 2,387 |
| E – Non-compliant | 3,109 | 3,859 | 3,358 |

## References

1. Myléus A, Ivarsson A, Webb C, Danielsson L, Hernell O, Högberg L, Karlsson E, Lagerqvist C, Norström F, Rosén A *et al*: **Celiac disease revealed in 3% of Swedish 12-year-olds born during an epidemic**. *J Pediatr Gastroenterol Nutr* 2009, **49**(2):170-176.

2. Hershcovici T, Leshno M, Goldin E, Shamir R, Israeli E: **Cost effectiveness of mass screening for coeliac disease is determined by time-delay to diagnosis and quality of life on a gluten-free diet**. *Aliment Pharmacol Ther* 2010, **31**(8):901-910.

3. Rostom A, Murray JA, Kagnoff MF: **American Gastroenterological Association (AGA) Institute Technical Review on the Diagnosis and Management of Celiac Disease**. *Gastroenterology* 2006, **131**(6):1981-2002.

4. Webb C, Halvarsson B, Norström F, Myléus A, Carlsson A, Danielsson L, Högberg L, Ivarsson A, Karlsson E, Stenhammar L *et al*: **Accuracy in celiac disease diagnostics by controlling the small-bowel biopsy process**. *J Pediatr Gastroenterol Nutr* 2011, **52**(5):549-553.

5. Ivarsson A, Persson LÅ, Nyström L, Ascher H, Cavell B, Danielsson L, Dannaeus A, Lindberg T, Lindquist B, Stenhammar L *et al*: **Epidemic of coeliac disease in Swedish children**. *Acta Paediatr* 2000, **89**(2):165-171.

6. Namatovu F, Sandström O, Olsson C, Lindkvist M, Ivarsson A: **Celiac disease risk varies between birth cohorts, generating hypotheses about causality: evidence from 36 years of population-based follow-up**. *BMC Gastroenterol* 2014, **14**(1):59.

7. Hall NJ, Rubin G, Charnock A: **Systematic review: adherence to a gluten-free diet in adult patients with coeliac disease**. *Aliment Pharmacol Ther* 2009, **30**(4):315-330.

8. Myléus A, Reilly NR, Green PHR: **Rate, Risk Factors, and Outcomes of Nonadherence in Pediatric Patients With Celiac Disease: A Systematic Review**. *Clin Gastroenterol Hepatol* 2020, **18**(3):562-573.

9. Viljamaa M, Collin P, Huhtala H, Sievanen H, Mäki M, Kaukinen K: **Is coeliac disease screening in risk groups justified? A fourteen-year follow-up with special focus on compliance and quality of life**. *Aliment Pharmacol Ther* 2005, **22**(4):317-324.

10. Norström F, Lindholm L, Sandström O, Nordyke K, Ivarsson A: **Delay to celiac disease diagnosis and its implications for health-related quality of life**. *BMC Gastroenterol* 2011, **11**(1):118.

11. Leffler DA, Dennis M, George JBE, Jamma S, Magge S, Cook EF, Schuppan D, Kelly CP: **A Simple Validated Gluten-Free Diet Adherence Survey for Adults With Celiac Disease**. *Clin Gastroenterol Hepatol* 2009, **7**(5):530-536.

12. Johansson K, Norström F, Nordyke K, Myléus A: **Celiac Dietary Adherence Test simplifies Determining Adherence to a Gluten-free Diet in Swedish Adolescents**. *J Pediatr Gastroenterol Nutr* 2019, **69**(5):575-580.

13. Tio M, Cox MR, Eslick GD: **Meta-analysis: coeliac disease and the risk of all-cause mortality, any malignancy and lymphoid malignancy**. *Aliment Pharmacol Ther* 2012, **35**(5):540-551.

14. Ludvigsson JF, Montgomery SM, Ekbom A, Brandt L, Granath F: **Small-intestinal histopathology and mortality risk in celiac disease**. *JAMA* 2009, **302**(11):1171-1178.

15. Lebwohl B, Green PHR, Söderling J, Roelstraete B, Ludvigsson JF: **Association Between Celiac Disease and Mortality Risk in a Swedish Population**. *JAMA* 2020, **323**(13):1277-1285.

16. Godfrey JD, Brantner TL, Brinjikji W, Christensen KN, Brogan DL, Van Dyke CT, Lahr BD, Larson JJ, Rubio-Tapia A, Melton LJ *et al*: **Morbidity and mortality among older individuals with undiagnosed celiac disease**. *Gastroenterology* 2010, **139**(3):763-769.

17. Lohi S, Mäki M, Rissanen H, Knekt P, Reunanen A, Kaukinen K: **Prognosis of unrecognized coeliac disease as regards mortality: a population-based cohort study**. *Ann Med* 2009, **41**(7):508-515.

18. Abdul Sultan A, Crooks CJ, Card T, Tata LJ, Fleming KM, West J: **Causes of death in people with coeliac disease in England compared with the general population: a competing risk analysis**. *Gut* 2015, **64**(8):1220-1226.

19. Holmes GKT, Muirhead A: **Mortality in coeliac disease: a population-based cohort study from a single centre in Southern Derbyshire, UK**. *Bmj Open Gastroenterology* 2018, **5**(1).

20. Quarpong W, Card TR, West J, Solaymani-Dodaran M, Logan RF, Grainge MJ: **Mortality in people with coeliac disease: Long-term follow-up from a Scottish cohort**. *United European Gastroenterol J* 2019, **7**(3):377-387.

21. Johnston SD, Watson RG, McMillan SA, Sloan J, Love AH: **Prevalence of coeliac disease in Northern Ireland**. *Lancet* 1997, **350**(9088):1370.

22. Catassi C, Fabiani E, Ratsch IM, Coppa GV, Giorgi PL, Pierdomenico R, Alessandrini S, Iwanejko G, Domenici R, Mei E *et al*: **The coeliac iceberg in Italy. A multicentre antigliadin antibodies screening for coeliac disease in school-age subjects**. *Acta Paediatr Suppl* 1996, **412**:29-35.

23. Catassi C, Ratsch IM, Fabiani E, Rossini M, Bordicchia F, Candela F, Coppa GV, Giorgi PL: **Coeliac disease in the year 2000: exploring the iceberg**. *Lancet* 1994, **343**(8891):200-203.

24. Corazza GR, Andreani ML, Biagi F, Corrao G, Pretolani S, Giulianelli G, Ghironzi G, Gasbarrini G: **The smaller size of the 'coeliac iceberg' in adults**. *Scand J Gastroenterol* 1997, **32**(9):917-919.

25. Norström F, Sandström O, Lindholm L, Ivarsson A: **A gluten-free diet effectively reduces symptoms and health care consumption in a Swedish celiac disease population**. *BMC Gastroenterol* 2012, **12**(1):125.
